# 

**Published:** 1870-05

**Authors:** 


### CONTENTS.

ORIGINAL COMMUNICATIONS.

The Proper Basis of Dental Charges... 197
Anæsthesia... 203
The Present Status of the Dental Profession... 209
The Past and Present of Dentistry... 215
Nitrous Oxide Gas... 219
The Brooklyn Dental Infirmary... 221
Teeth of the Ancients... 223

SELECTIONS.

Physiology and Chemistry of Old Age... 227
Nursing Sore Mouth... 234
Programme of the Mad River Valley Dental Society... 236
Thermometer at Nashville... 236

EDITORIAL.

Report of Nitrous Oxyd Cases... 237

The Mallet... 238

Thoughts for Young Men... 240

